# Sar1 Interacts with Sec23/Sec24 and Sec13/Sec31 Complexes: Insight into Its Involvement in the Assembly of Coat Protein Complex II in the Microsporidian Nosema bombycis

**DOI:** 10.1128/spectrum.00719-22

**Published:** 2022-10-27

**Authors:** Fuzhen Sun, Runpeng Wang, Ping He, Erjun Wei, Qiang Wang, Xudong Tang, Yiling Zhang, Feng Zhu, Zhongyuan Shen

**Affiliations:** a School of Biotechnology, Jiangsu University of Science and Technology, Zhenjiang, China; b Institute of Sericulture, Chinese Academy of Agricultural Sciences, Zhenjiang, China; c College of Life Sciences, Zaozhuang University, Zhenjiang, China; Centro de Investigaciones Biologicas CSIC

**Keywords:** microsporidia, *Nosema bombycis*, endoplasmic reticulum, coat protein complex II, Sar1

## Abstract

Microsporidia, as unicellular eukaryotes, also have an endomembrane system for transporting proteins, which is essentially similar to those of other eukaryotes. In eukaryotes, coat protein complex II (COPII) consists of Sar1, Sec23, Sec24, Sec13, and Sec31 and mediates protein transport from the endoplasmic reticulum (ER) to the Golgi apparatus. Sar1 is the central player in the regulation of coat protein complex II vesicle formation in the endoplasmic reticulum. In this study, we successfully cloned the *NbSar1*, *NbSec23-1*, *NbSec23-2*, *NbSec24-1*, *NbSec24-2*, *NbSec13*, *NbSec31-1*, and *NbSec31-2* genes and prepared NbSar1 polyclonal antibody. We found that NbSar1 was localized mainly in the perinuclear cytoplasm of Nosema bombycis by immunofluorescence analysis (IFA). Yeast two-hybrid assays demonstrated that NbSar1 interacts with NbSec23-2, NbSec23-2 interacts with NbSec24-1 or NbSec24-2, NbSec23-1 interacts with NbSec31, and NbSec31 interacts with NbSec13. Moreover, the silencing of *NbSar1* by RNA interference resulted in the aberrant expression of *NbSar1*, *NbSec23-1*, *NbSec24-1*, *NbSec24-2*, *NbSec13*, *NbSec31-1*, and *NbSec31-2* and significantly inhibited the proliferation of N. bombycis. Altogether, these findings indicated that the subunits of coat protein complex II work together to perform functions in the proliferation of *N. bombycis* and that NbSar1 may play a crucial role in coat protein complex II vesicle formation.

**IMPORTANCE** As eukaryotes, microsporidia have retained the endomembrane system for transporting and sorting proteins throughout their evolution. Whether the microsporidia form coat protein complex II (COPII) vesicles to transport cargo proteins and whether they play other roles besides cargo transport are not fully explained at present. Our results showed that NbSar1, NbSec23-1/NbSec23-2, NbSec24-1/NbSec24-2, NbSec13, and NbSec31 might be assembled to form COPII in the ER of *N. bombycis*, and the functions of COPII are also closely related to the proliferation of *N. bombycis*, this may be a new target for the prevention of pébrine disease of the silkworm.

## INTRODUCTION

**FIG 1:**
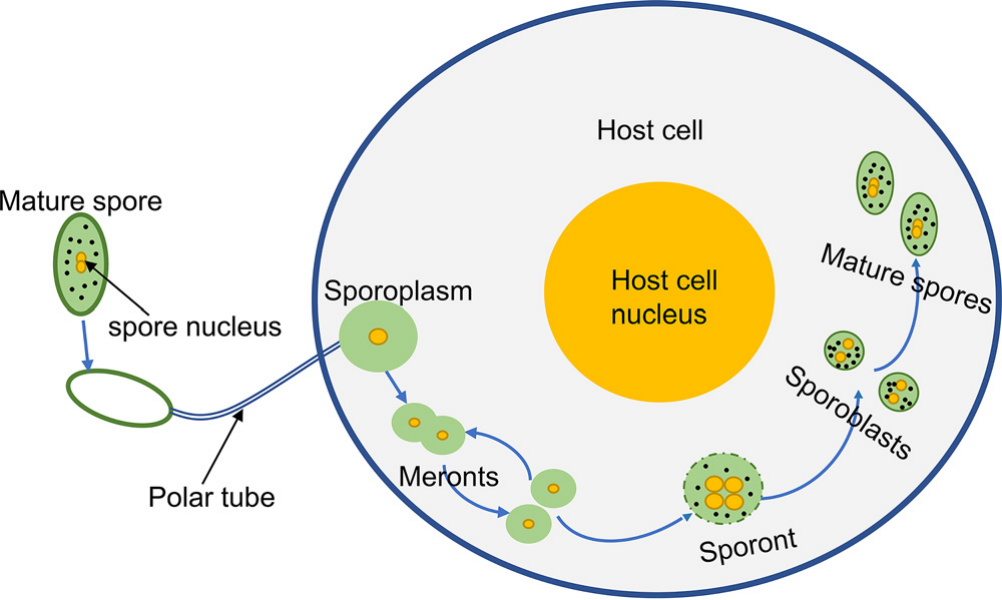
Diagrammatic illustration of the developmental cycle of *N. bombycis*. The life cycle of *N. bombycis* is divided into an infective phase, a proliferative phase, and a sporogonic phase. In the infective phase, the mature spore germinates and injects the sporoplasm into the host cell. In the proliferative phase, the meront proliferates by binary fission or multiple fissions in the host cell. In the sporogonic phase, the sporont with four nuclei divides into two sporoblasts, and the sporoblasts further develop into mature spores with thick walls.

Microsporidia are obligate intracellular parasites that can infect a wide range of vertebrates and invertebrates, including humans (1). Microsporidia are classified into 200 genera including more than 1,400 species (2). Nosema bombycis, the pathogen causing pébrine disease in the silkworm, was discovered in the silkworm by Naegeli in 1857. Pébrine is transmitted both horizontally and vertically to the silkworm, resulting in great economic losses to the silkworm industry (3, 4). The life cycle of N. bombycis is divided into three phases: the infective phase, the proliferative phase, and the sporogonic phase (Fig. 1).

Eukaryotic cells maintain life activities by synthesizing proteins and lipids for secretion and transport to specific organelles and plasma membranes. Secretory and membrane proteins are synthesized in the ribosome and transferred to the compartment of the endoplasmic reticulum (ER) or integrated into the ER membrane. The transport of prefolded proteins from the ER to the Golgi apparatus is performed by coat protein complex II (COPII)-mediated vesicular transport (5). COPII consists of five proteins that are highly conserved in eukaryotes: the small G protein Sar1, the inner coat proteins Sec23 and Sec24, and the outer coat proteins Sec13 and Sec31 (6). Sar1 is inserted into the ER membranes in a Sar1-GTP-activated form to recruit the Sec23-Sec24 complex through binding Sec23, eventually forming the inner layer of COPII vesicles (7, 8). The Sec23-Sec24 complex is bent and positively charged on one side, which facilitates the binding of the complex to the ER membrane (8), while Sec23 recruits the Sec13-Sec31 complex to form the caged outer layer of COPII vesicles by binding to the proline-rich domain at the C terminus of Sec31 and driving the deformation of the ER membrane (9). The role of Sar1, a small GTPase, in vesicle budding has been extensively studied in Saccharomyces cerevisiae. Sar1 is involved in vesicle formation only when it is activated by the ER membrane protein Sec12 (10, 11). Therefore, Sar1 is a central player in the regulation of COPII vesicle formation in the ER.

Microsporidia exhibit extreme compaction in genome size due to their intracellular parasitic lifestyle, resulting in the loss of genes associated with their metabolic pathways and a high degree of organelle simplification (12–17). As eukaryotes, microsporidia have retained the endomembrane system for transporting and sorting proteins. Membrane polymers of the Golgi apparatus were observed in the early sporont and aggregated around flattened vesicles of the ER near the perinuclear lumen (18, 19). The tubular structures of the Golgi apparatus have been found in sporont and mature spores of microsporidia (20, 21), and the tubular structure is connected to the perinuclear lumen, ER, plasma membrane, and polar tube (22). Expectedly, the five subunits of COPII, Sar1, Sec23, Sec24, Sec13, and Sec31, are present in *N. bombycis* according to genomic analyses. Previously, it was reported that the spore wall and polar tube proteins were transported from the ER to the plasma membrane through tubular networks (TNs) in microsporidia, which does not require the involvement of vesicles produced by COPI or COPII (22). But studies on COPII of microsporidia have been rare until now. Whether the microsporidia form COPII vesicles to transport cargo proteins and whether COPII of microsporidia plays various roles besides cargo transport are not fully explained at present. In order to understand the mechanism of COPII in the proliferation of *N. bombycis*, we studied the regulatory and interaction relationships among *N. bombycis* Sar1 (NbSar1), NbSec23, NbSec24, NbSec13, and NbSec31 in the microsporidian *N. bombycis* for the first time. We have found that NbSar1 is localized in the ER of *N. bombycis* and interacts with NbSec23-2 and that NbSec23-2 interacts with NbSec24-1/NbSec24-2, NbSec23-1 interacts with NbSec31, and NbSec31 interacts with NbSec13. Moreover, RNAi (RNA interference) on *NbSar1* resulted in the abnormal expression of *NbSec23-1*, *NbSec24-1*/*NbSec24-2*, *NbSec31-1*/*NbSec31-2*, and *NbSec13*. These findings indicated that NbSar1, NbSec23-1/NbSec23-2, NbSec24-1/NbSec24-2, NbSec13, and NbSec31 might be assembled to form COPII in the ER of *N. bombycis*.

## RESULTS

### Cloning and expression of the *NbSar1* gene and sequence analysis.

**FIG 2:**
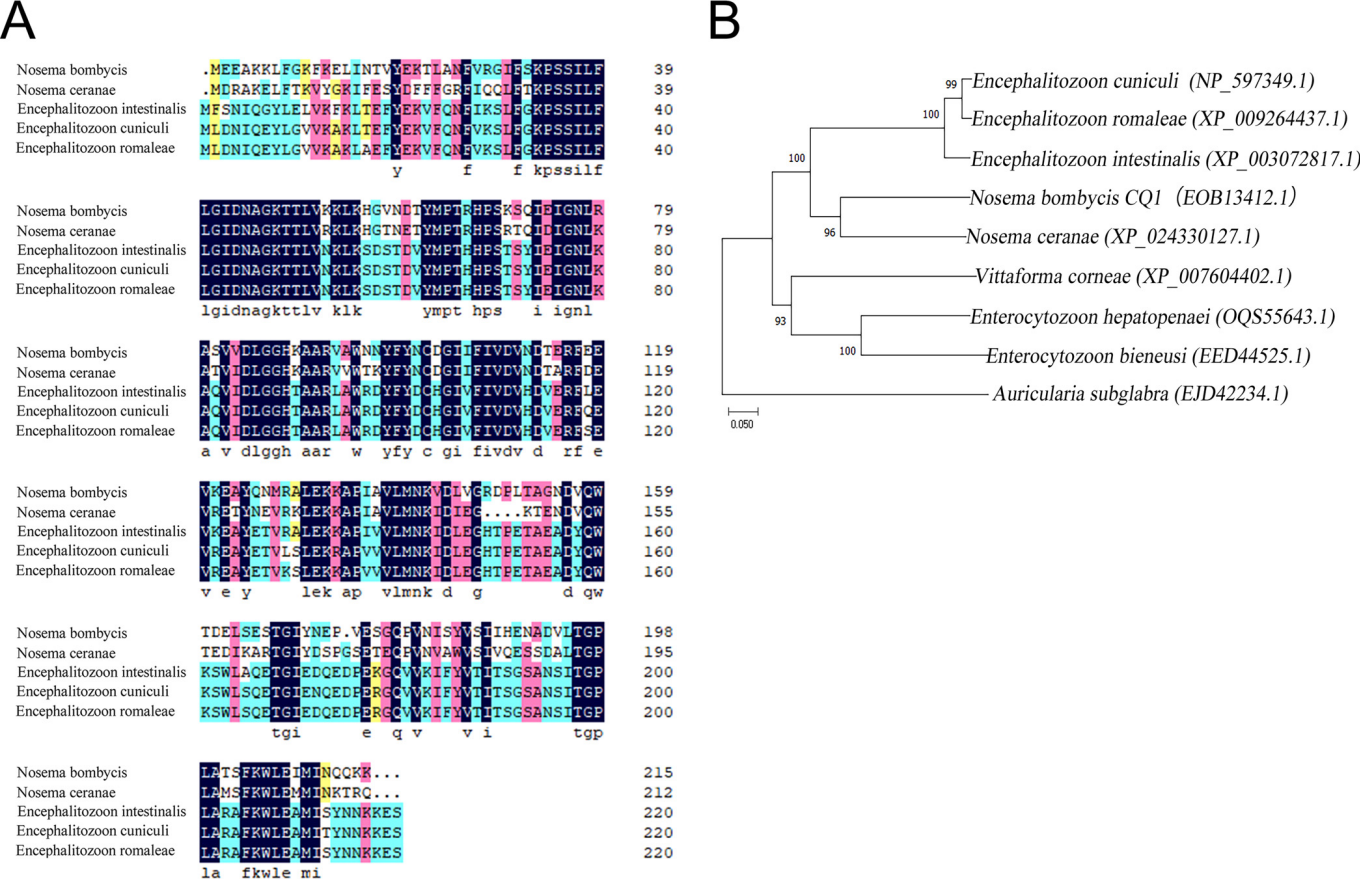
Bioinformatics analysis of Sar1. (A) Amino acid alignment of Sar1 proteins from *N. bombycis* and other microsporidian species. (B) Phylogenetic tree of Sar1 proteins from *N. bombycis* and other microsporidian species. Protein sequences were aligned using ClustalW. The maximum likelihood (ML) tree was constructed using MEGA 6.0 software, and bootstrap analyses were performed by employing Jones-Taylor-Thornton (JTT) model-based distance matrices generated from 1,000 resamplings of the alignments. Values at the branches indicate bootstrap support.

PCR amplification and sequencing results showed that the *NbSar1* gene contains a complete open reading frame (ORF) of 648 bp in length that encodes 215 amino acids. Homologous multiple-sequence alignment revealed that NbSar1 is highly homologous to other microsporidian Sar1 proteins, with a homology of >35%, and that the amino acid similarities between NbSar1 and the Sar1 proteins of Nosema ceranae (GenBank accession number XP_024330127.1), Encephalitozoon intestinalis (accession number XP_003072817.1), Encephalitozoon cuniculi (accession number NP_597349.1), and Encephalitozoon romaleae (accession number XP_009264437.1) were 63.89%, 57.01%, 56.56%, and 56.56%, respectively (Fig. 2A). Phylogenetic analysis results showed that *N. bombycis* and N. ceranae were clustered into the same branch (Fig. 2B), indicating that they were closely related to each other.

**FIG 3:**
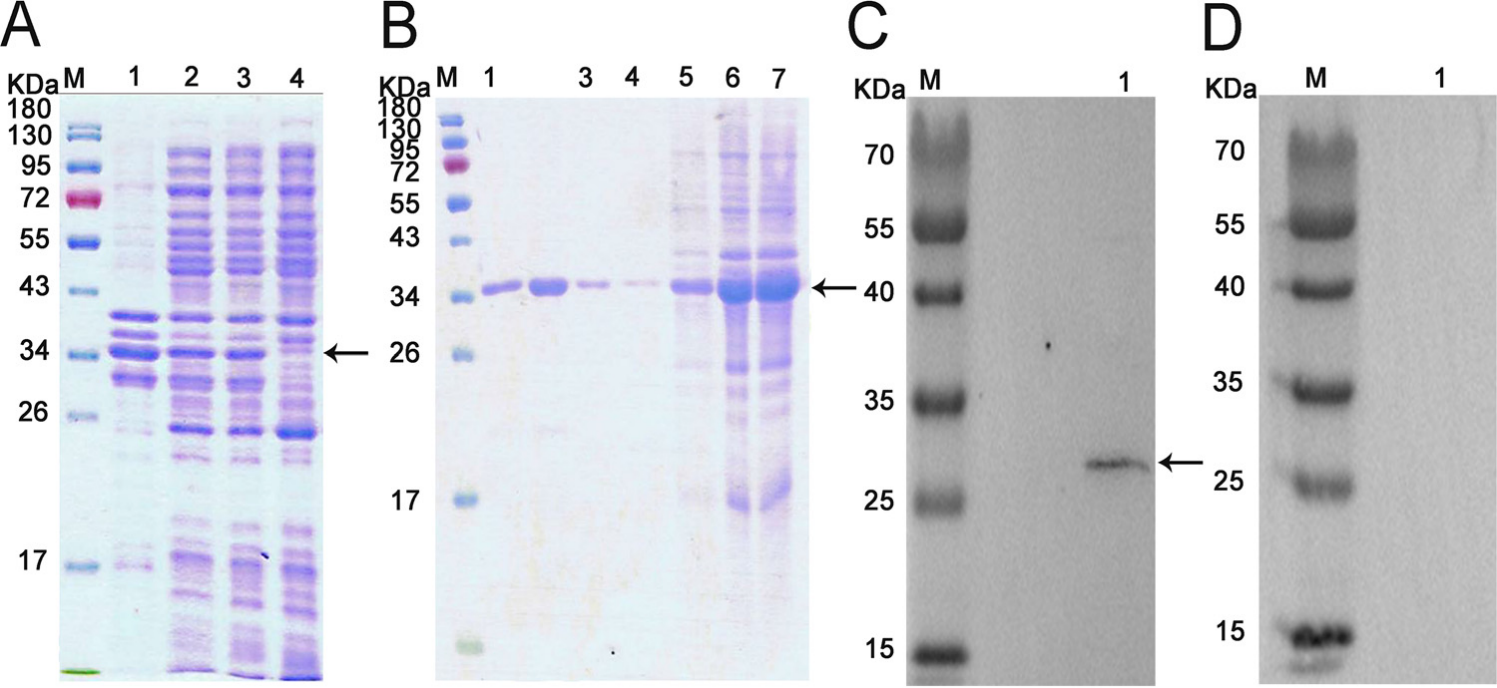
Expression, purification, and Western blot analysis of recombinant NbSar1. (A) SDS-PAGE analysis of the expressed recombinant protein. Lane M, protein marker; lane 1, the precipitate of induced recombinant bacteria; lane 2, the supernatant of induced recombinant bacteria; lane 3, recombinant bacteria induced with 0.5 mM IPTG at 37°C for 4 h; lane 4, recombinant bacteria without induction. (B) SDS-PAGE analysis of the purified fusion protein. Lane M, protein marker; lane 1, elution with 500 mmol/L imidazole; lane 2, elution with 200 mmol/L imidazole; lane 3, elution with 50 mmol/L imidazole; lane 4, elution with 20 mmol/L imidazole; lane 5, elution with 10 mmol/L imidazole; lane 6, flowthrough; lane 7, unpurified recombinant NbSar1 protein. (C) Specificity analysis of NbSar1 antibody. Lane M, protein marker; lane 1, total protein of an *N. bombycis* mature spore. (D) Preimmune serum control. Lane M, protein marker; lane 1, total protein of an *N. bombycis* mature spore. The arrows indicate NbSar1 protein.

SDS-PAGE results showed that the molecular weight of the NbSar1 recombinant protein was about 34 kDa, which included the NbSar1 protein and the His tag. Ultrasonic fragmentation showed that the NbSar1 protein was present mainly in the form of inclusion bodies in isopropyl-β-d-thiogalactopyranoside (IPTG)-induced bacteria (Fig. 3A). Purified recombinant proteins were obtained by elution with imidazole at different concentrations (Fig. 3B). Western blotting indicated that a 28-kDa protein band was detected in the total protein of *N. bombycis*, which corresponds to the predicted size of the NbSar1 amino acid sequence (Fig. 3C). Preimmune serum, which served as the negative control, did not detect the target protein (Fig. 3D).

### Subcellular localization of NbSar1 in different developmental phases of *N. bombycis*.

**FIG 4:**
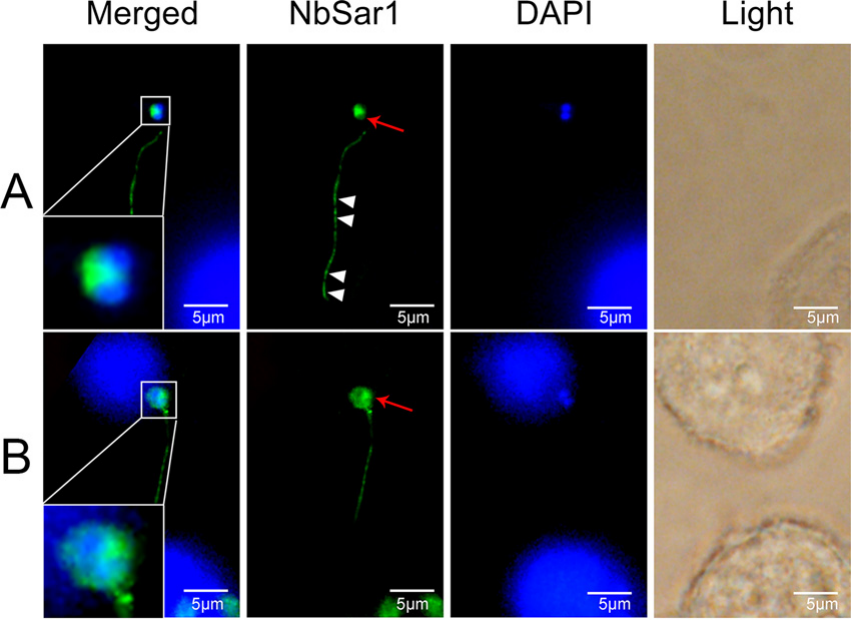
Subcellular localization of NbSar1 during intracellular infection. (A) Germinating spore. The white arrowheads indicate that the green fluorescent signals are distributed in the polar tube, and the red arrows indicate that the green fluorescent signals are distributed in the cytoplasm of the sporoplasm. (B) After spore germination. The red arrows indicate that the green fluorescent signals are distributed mainly in the perinuclear cytoplasm of the sporoplasm. DAPI (blue) was used to stain the nuclei of host cells and *N. bombycis*. NbSar1 antibody was coupled with Alexa Fluor 488 (green). BmN cells were infected with *N. bombycis* for 7 days.

**FIG 5:**
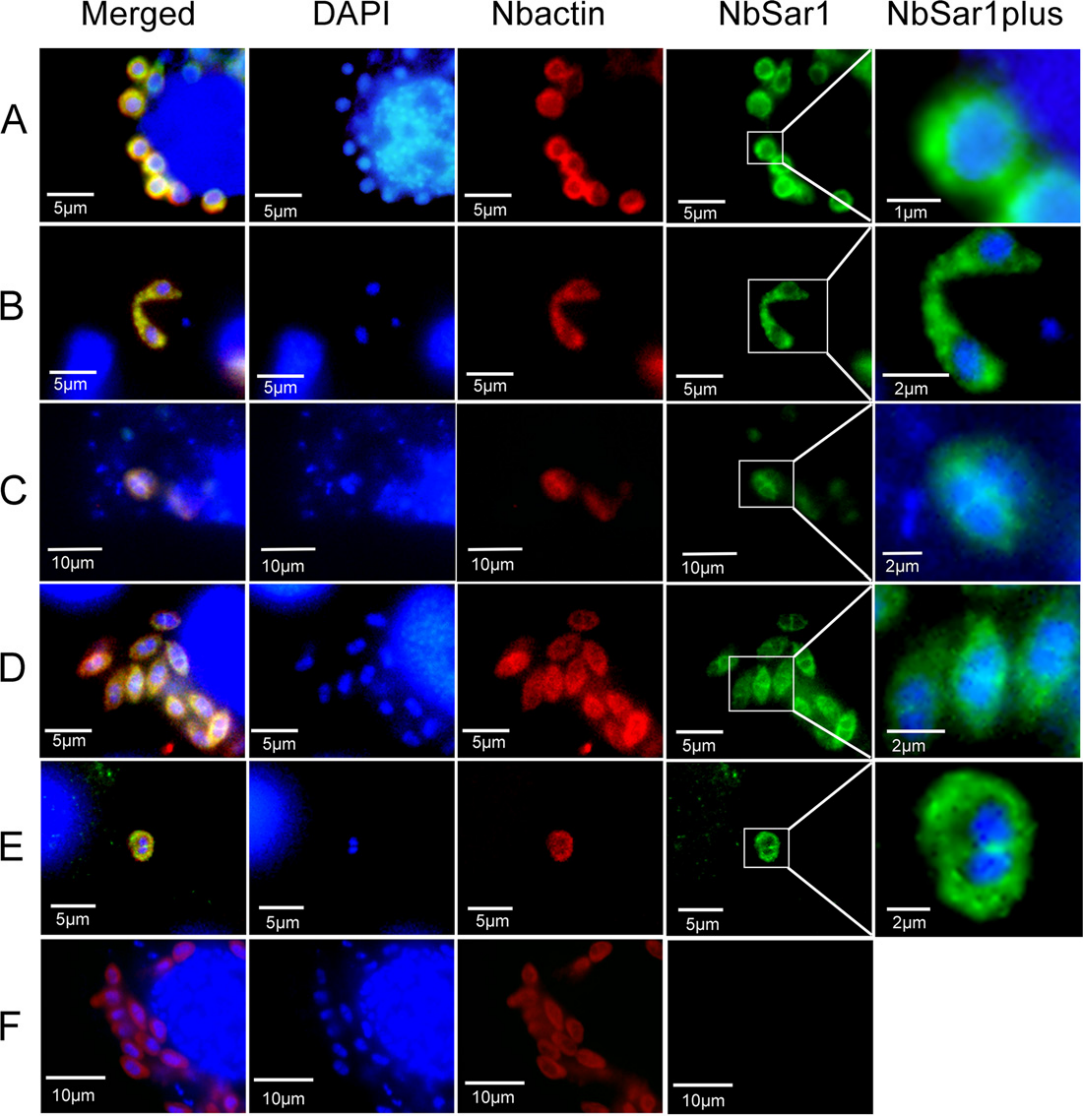
Subcellular colocalization of NbSar1 and *N. bombycis* actin in *N. bombycis*. (A) Sporoplasm. (B) Meront. (C) Sporont. (D) Sporoblasts. (E) Sporoblast nearing maturation into a spore. (F) Preimmune serum. DAPI (blue) was used to stain the nuclei of host cells and *N. bombycis*. NbSar1 antibody was coupled with Alexa Fluor 488 (green), and *N. bombycis* actin (Nbactin) antibody was coupled with Cy5 (red). BmN cells were infected with *N. bombycis* for 7 days.

To investigate the localization of NbSar1 in the intracellular phase of *N. bombycis*, NbSar1 antibodies, marked with green fluorescence, were used to perform immunofluorescence analysis (IFA). In the infective phase, when the polar tube was everted from the germinating spore and the two unfused nuclei were expelled outside the spore, the green fluorescent signals were distributed mainly in the perinuclear cytoplasm of the sporoplasm and in the polar tube (Fig. 4A). After the contents of the *N. bombycis* spore were completely expelled from the germinated spore and injected into the new host cell, the green fluorescent signals were distributed mainly in the perinuclear cytoplasm of the sporoplasm (Fig. 4B). When the two nuclei of the invaded sporoplasm fused into one nucleus, the green and red fluorescent signals (*N. bombycis* actin antibodies were marked with red fluorescence as a reference label) were distributed in the perinuclear cytoplasm of the sporoplasm (Fig. 5A). In the proliferative phase, when *N. bombycis* was undergoing rapid proliferation, the green and red fluorescent signals were distributed mainly in the cytoplasm (Fig. 5B). In the sporogonic phase, green and red fluorescent signals were distributed in the perinuclear region of sporont with four nuclei (Fig. 5C). Sporoblasts are cells derived from the final division of the sporont. In the sporoblasts, the green and the red fluorescent signals were distributed mainly in the perinuclear region (Fig. 5D). When the *N. bombycis* cell neared maturation, the green and the red fluorescent signals were distributed mainly in the cytoplasm (Fig. 5E). No green fluorescent signals were observed in the preimmune serum group (Fig. 5F). These results suggested that NbSar1 exists mainly in the perinuclear region of *N. bombycis* throughout its life cycle.

### Interactions between NbSar1 and NbSec23-2, NbSec23-2 and NbSec24, NbSec23-1 and NbSec31-1, and NbSec31-1 and NbSec13.

**FIG 6:**
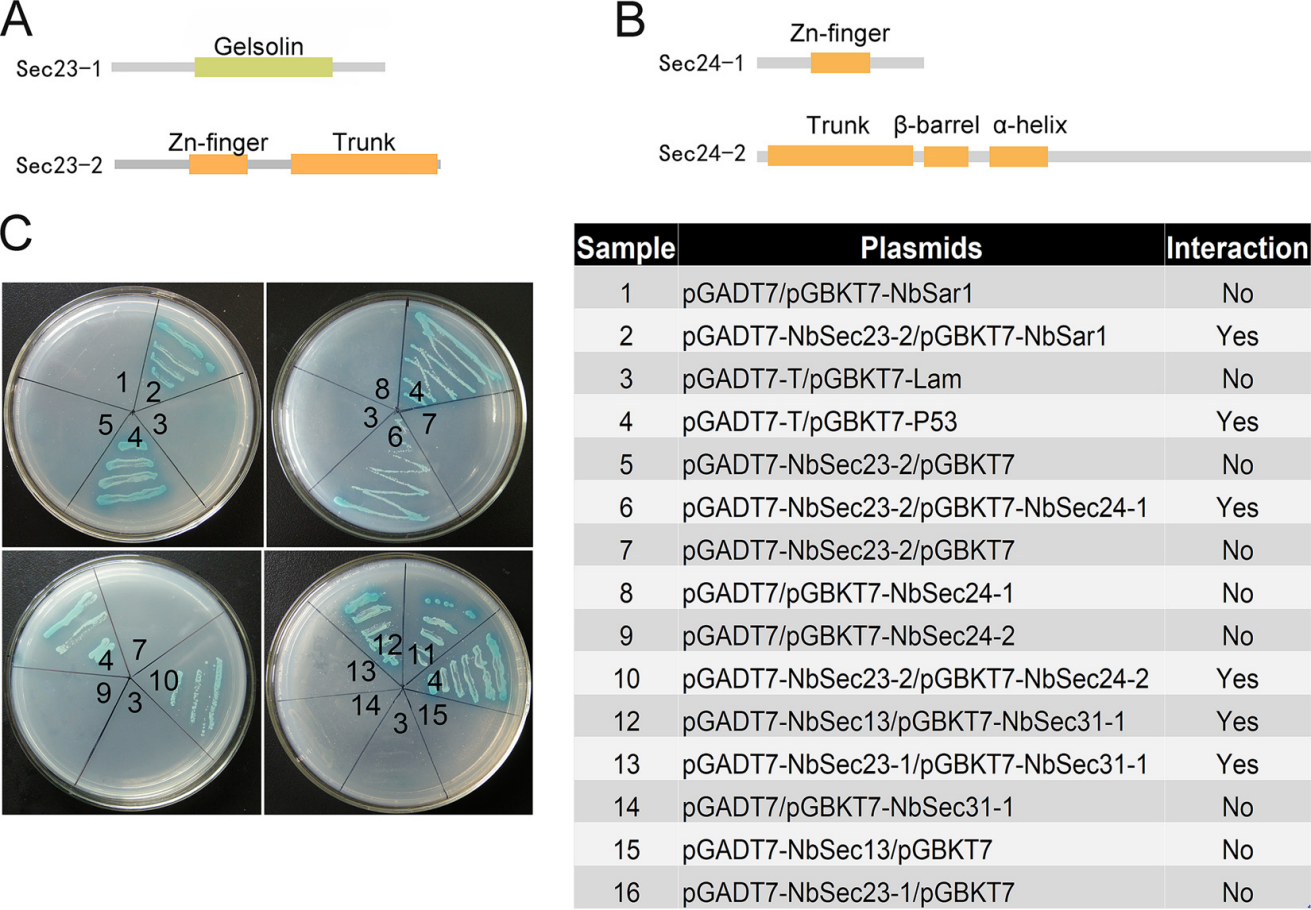
Domains and interactions between subunits of COPII. (A) Domains of NbSec23-1 and NbSec23-2. (B) Domains of NbSec24-1 and NbSec24-2. (C) Yeast two-hybrid analysis of the interactions between NbSar1 and NbSec23-2, NbSec23-2 and NbSec24-1/NbSec24-2, NbSec23-1 and NbSec31-1, and NbSec13 and NbSec31-1. The interaction of pGADT7-T and pGBKT7-P53 was used as the positive control, and the interaction of pGADT7-T and pGBKT7-lam was used as the negative control.

In this study, cloning results showed that *NbSec23-1* (GenBank accession number EOB11408.1), *NbSec23-2* (accession number EOB12942.1), *NbSec24-1* (accession number EOB13378.1), *NbSec24-2* (accession number EOB11737.1), *NbSec13* (accession number EOB15402.1), *NbSec31-1* (accession number EOB13833.1), and *NbSec31-2* (accession number EOB13782.1) contain complete ORFs of 540 bp, 648 bp, 648 bp, 1,905 bp, 825 bp, 2,463 bp, and 2,721 bp, respectively. The domain prediction results showed that *NbSec23-1* contains only the gelsolin domain; *NbSec23-2* contains the trunk domain and the Zn finger domain (Fig. 6A); *NbSec24-1* contains only the Zn finger domain; and *NbSec24-2* contains the trunk domain, the β-barrel domain, and the α-helix domain (Fig. 6B). Sequence alignment results showed that *NbSec31-2* includes the complete sequence of *NbSec31-1*. The interactions between NbSar1 and NbSec23-2, NbSec23-2 and NbSec24-1/NbSec24-2, NbSec23-1 and NbSec31-1, and NbSec31-1 and NbSec13 were examined by a yeast two-hybrid assay. The yeast two-hybrid assay showed that activating domain (AD)–NbSec23-2 and BK-NbSar1, AD–NbSec23-2 and Binding Domain (BK)–NbSec24-1/BK–NbSec24-2, AD–NbSec23-1 and BK–NbSec31-1, and AD-NbSec13 and BK–NbSec31-1 grew successfully on synthetic dropout (SD)−Ade/−His/−Leu/−Trp selective medium with 5-Bromo-4-chloro-3-indoxyl-α-d-galactopyranoside (X-α-gal) (Fig. 6C). The above results proved that NbSar1 interacts with NbSec23-2, NbSec23-2 interacts with NbSec24-1/NbSec24-2, NbSec23-1 interacts with NbSec31-1, and NbSec31-1 interacts with NbSec13, which is consistent with previous studies showing that the assembly of COPII vesicles depends on the interactions among Sar1, Sec23, Sec24, Sec13, and Sec31 and that the trunk domain of Sec23 interacts with Sec24 to form the Sec23-Sec24 complex, Sec13 interacts with Sec31 to form the Sec13-Sec13 complex, Sar1 recruits the Sec23-Sec24 complex through interaction with the trunk domain of Sec23, and the gelsolin domain of Sec23 recruits the Sec13-Sec31 complex through interaction with the proline-rich domain at the C terminus of Sec31 (7, 23–26). Our findings suggested that NbSar1, NbSec23, NbSec24, NbSec13, and NbSec31 could assemble a complex through interaction.

### RNAi on *NbSar1* downregulated the expression of *NbSec23-1*, *NbSec31-1*, and *NbSec31-2* but upregulated the expression of *NbSec24-1*, *NbSec24-2*, and *NbSec13*.

**FIG 7:**
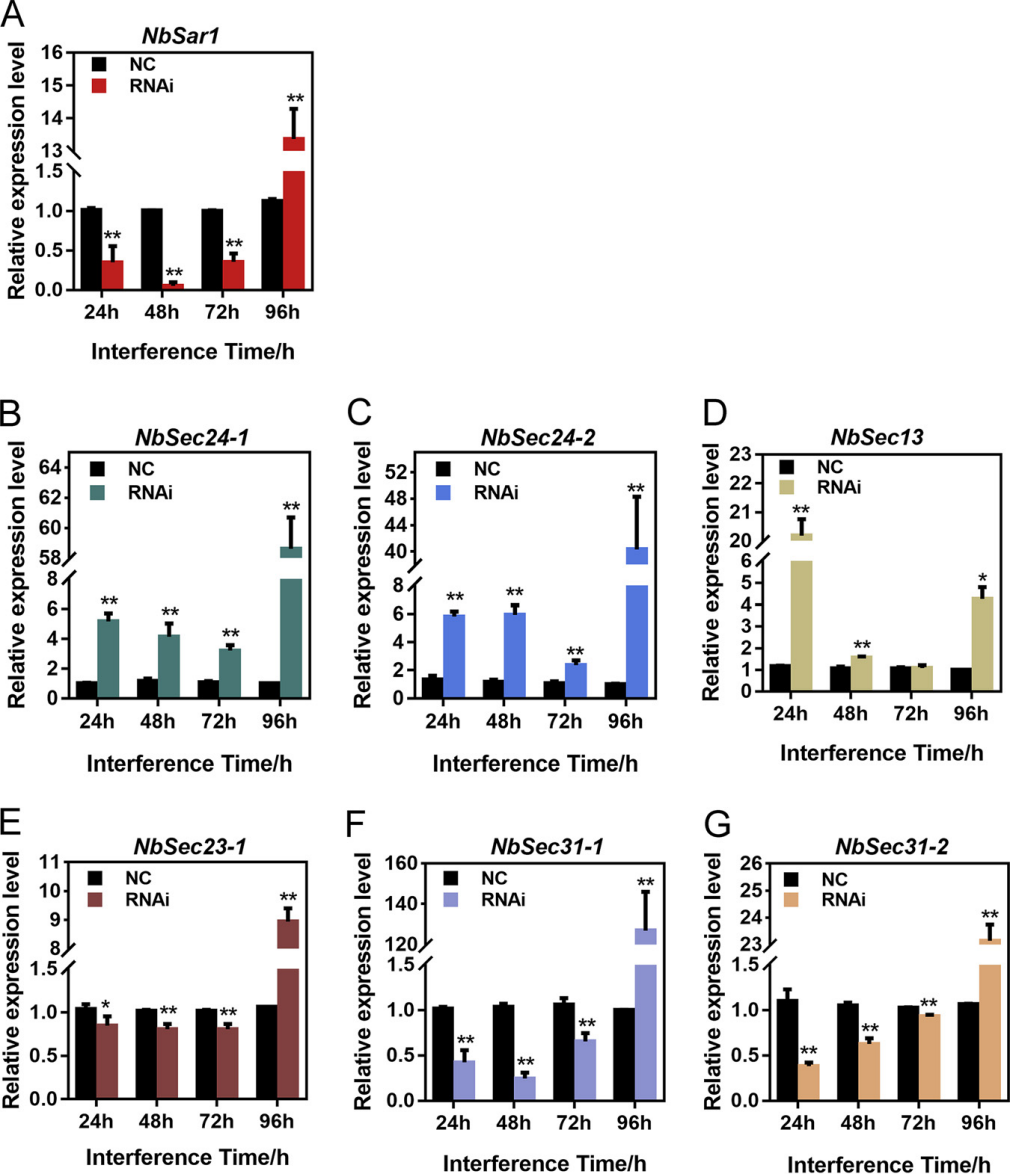
Effect of the knockdown of *NbSar1* on the expression of subunits of COPII. (A) Relative expression level of the *NbSar1* gene after RNAi. (B) Relative expression level of the *NbSec24-1* gene after RNAi. (C) Relative expression level of the *NbSec24-2* gene after RNAi. (D) Relative expression level of the *NbSec13* gene after RNAi. (E) Relative expression level of the *NbSec23-1* gene after RNAi. (F) Relative expression level of the *NbSec31-1* gene after RNAi. (G) Relative expression level of the *NbSec31-2* gene after RNAi. NC, control group; RNAi, RNAi-NbSar1 group. Asterisks represent statistically significant differences (*, *P < *0.05; **, *P* < 0.01). The error bars represent the standard deviations from three independent repeats.

To assess the interference effects of small interfering RNA (siRNA) on *NbSar1* expression, silkworms infected with *N. bombycis* were injected with Sar1 siRNA, while the control group was injected with nonsense siRNA. Quantitative PCR (qPCR) was performed to analyze the expression pattern of NbSar1. As a result, the relative expression level of *NbSar1* was significantly decreased following siRNA treatment for 24 h, 48 h, and 72 h, whereas it was significantly increased at 96 h (Fig. 7A). This proved that RNAi on *NbSar1* can effectively depress the expression of *NbSar1* at 24 h, 48 h, and 72 h.

To determine the effect of knocking down *NbSar1* on the expression of COPII subunits, the *N. bombycis* β-tubulin gene served as the reference gene to analyze the expression patterns of *NbSec23-1*, *NbSec24-1*, *NbSec24-2*, *NbSec13*, *NbSec31-1*, and *NbSec31-2* by qPCR. The results showed that *NbSec23-1*, *NbSec31-1*, and *NbSec31-2* were significantly downregulated (Fig. 7B, E, and F) whereas *NbSec24-1* and *NbSec24-2* were significantly upregulated following siRNA treatment for 24 h, 48 h, and 72 h (Fig. 7C), *NbSec13* was significantly upregulated following siRNA treatment for 24 h and 48 h (Fig. 7D). However, *NbSec23-1*, *NbSec24-1*, *NbSec24-2*, *NbSec13*, *NbSec31-1*, and *NbSec31-2* were all significantly upregulated 96 h after siRNA treatment (Fig. 7B to F). These results suggested that *NbSar1*, *NbSec23-1*, *NbSec24-1*, *NbSec24-2*, *NbSec13*, *NbSec31-1*, and *NbSec31-2* may work together as a complex for the proliferation of *N. bombycis*.

### Knockdown of *NbSar1* suppressed the proliferation of *N. bombycis*.

**FIG 8:**
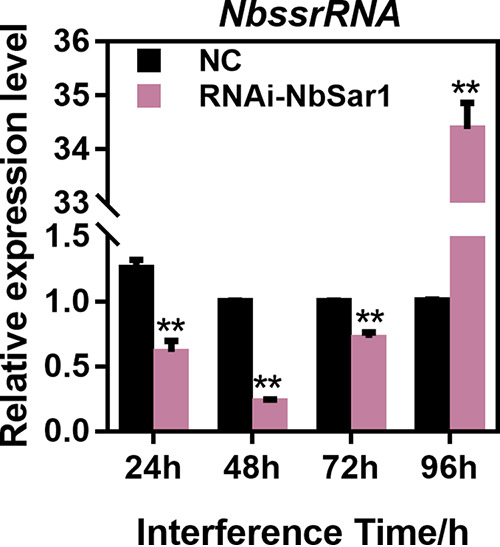
Knockdown of *NbSar1* inhibited the proliferation of *N. bombycis*. Shown are the relative expression levels of the *N. bombycis* ssrRNA gene after RNAi. NC, control group; RNAi, RNAi-NbSar1 group. Asterisks represent statistically significant differences (*, *P* < 0.05; **, *P* < 0.01). The error bars represent the standard deviations from three independent repeats.

To further explore the effect of knocking down the *NbSar1* gene on the proliferation of *N. bombycis*, the expression patterns of the small-subunit rRNA (ssrRNA) of *N. bombycis* were used to reflect the proliferation of *N. bombycis*. The effect of RNAi was analyzed by qPCR, with the *BmGAPDH* (Bombyx mori glyceraldehyde-3-phosphate dehydrogenase) gene serving as a reference gene. After the knockdown of *NbSar1*, the transcription level of *N. bombycis* ssrRNA was significantly downregulated in the RNAi-NbSar1 groups from 24 h to 72 h and then significantly upregulated at 96 h (Fig. 8). These results suggested that NbSar1 plays an important role in the proliferation of *N. bombycis*.

## DISCUSSION

Sar1, a small G protein, acts as a molecular switch for vesicular transport and is highly conserved in fungi, mammals, as well as plants. In this study, IFA was performed to determine the localization of Sar1 at different developmental stages of *N. bombycis*. The results showed that NbSar1 is distributed mainly in the perinuclear region of the cytoplasm and is clearly excluded from the nucleus in the intracellular phase of *N. bombycis*. These findings are consistent with those of previous studies showing that Sar1 was distributed along the perinuclear contour and in ER membranes in CHO-K1 cells (27) and that Sar1 of S. cerevisiae was located mainly around the periphery of the ER and in the perinuclear region (28, 29). In microsporidia, ultrastructure analysis showed the presence of ER tubular structures in the perinuclear region of Paranosema grylli (22), and the sporont of Berwaldia singularis contains more vesicles and a rough ER surrounding the nucleus (30). After spore germination, the newly ejected sporoplasm contains the outer membrane, vesicles, cytoplasm, and ER to which ribosomes are attached (18). These results indicated that the Sar1 may be localized to the ER of *N. bombycis*.

Interactions between NbSar1 and NbSec23-2, NbSec23-2 and NbSec24-1/NbSec24-2, NbSec23-1 and NbSec31-1, and NbSec31-1 and NbSec13 were successfully verified by yeast two-hybrid assays in this study. In yeast, Sec23 and Sec24 contain five distinct domains: the zinc finger domain, the trunk domain or α/β vWA (von Willebrand factor [vWF] type A domain), the β-barrel domain, the α-helix domain, and the gelsolin domain (7). Although the Sec23 proteins of E. cuniculi, Enterocytozoon hepatopenaei, Mitosporidium daphniae, and *N. ceranae* and the Sec24 proteins of E. intestinalis and Nosema granulosis contain all five distinct domains, the Sec23 and Sec24 proteins of other microsporidia such as Dictyocoela roeselum, Hamiltosporidium magnivora, *Amphiamblys* sp., and Enterospora canceri contain 1 to 4 domains, respectively. In *N. bombycis*, NbSec23-1 has only the gelsolin domain, and NbSec23-2 has the zinc finger domain and the trunk domain, while NbSec24-1 has only the zinc finger domain, and NbSec24-2 has the trunk domain, the β-barrel domain, and the α-helix domain. The multiple-sequence alignment showed that NbSec23/NbSec24 of *N. bombycis* has high similarity to those of other microsporidia (see Fig. S1 and S2 in the supplemental material). Moreover, the specific and efficient Sar1 recruitment to the ER membrane is accomplished by the guanine exchange factor (GEF) Sec12, whose role in the biogenesis of COPII is crucial (8). Sec12, which shows a high degree of sequence divergence in different species, is a type II transmembrane protein, and only the K-loop motif (GGGGxxxxGϕxN, where x indicates any amino acid, the ϕ indicates a hydrophobic residue) in Sec12 is highly conserved (31, 32). In Aspergillus nidulans, a protein with the Sec12 K-loop motif and a single-pass type II transmembrane is located on the ER and promotes specific nucleotide exchange on Sar1 as a GEF (GDP to GTP) of Sar1 (33). Although we have not found proteins containing the K-loop motif in *N. bombycis* by using PSI-BLAST, related proteins have been screened in some microsporidia, including *N. ceranae*, Nosema apis, Enterospora canceri, and so on. Previous research showed that the uniparental inheritance of microsporidia led to the evolution of two unusual traits: male killing and feminization of the host under the long-term coevolution of microsporidia and the host (34). Furthermore, alleles may be lost and novel genotypes could arise during the adaptation of microsporidia to a new host (35–37). These results indicated that the categories and numbers of domains of Sec23, Sec24, and relevant proteins in microsporidia displayed diversity, which may have resulted from the coevolution relationship between the microsporidium and its host.

It has been reported that the kinase inhibitor H89 affected the early assembly of COPII and ultimately inhibited vesicular stomatitis virus glycoprotein microsome export from the ER by blocking the membrane recruitment and activation of Sar1 (38). The silencing of mammalian *Sar1A* and *Sar1B* by siRNA caused a disruption of COPII assembly (39). In order to understand the regulatory role of NbSar1 in COPII assembly by microsporidia, knockdown of *NbSar1* was performed to assess the interference effect on the expression of other subunits of COPII. Interestingly, the expression levels of *NbSec23-1*, *NbSec31-1*, and *NbSec31-2* were significantly downregulated (Fig. 7C, E, and F), whereas the expression levels of *NbSec24-1*, *NbSec24-2*, and *NbSec13* were significantly upregulated (Fig. 7B and D). Nevertheless, whether a compensatory mechanism exists between Sec23 and Sec24 or between Sec13 and Sec31 is unknown. These findings shed new light: the knockdown of *NbSar1* suppresses the normal expression of *NbSec23*, *NbSec24*, *NbSec13*, and *NbSec31*. Previous studies found that the addition of the protein kinase inhibitor H89 leads to the inactivation of Sar1 and the accumulation of Sec23, Sar1, and GalT2 at the ER exit sites (ERESs) (27). The Sar1 temperature-sensitive allele has been successfully employed in some fungal models such as S. cerevisiae and A. nidulans (40–42). In the genetic model A. nidulans, the temperature inactivation of allele mutations of SarA leads to markedly reduced SarA levels and the aggregation of Sec23-green fluorescent protein (GFP) into very few spots per cell at high temperature, suggesting that these alleles cause temperature-dependent SarA misfolding. Furthermore, the SarA6 Ser186Pro substitution leads to the formation of apical balloons resembling specialized fungal structures at 37°C, which underscores the importance of the C-terminal α-helix for SarA function/stability (40). These studies exhibited results similar to those of our research: the inactivation or silencing of Sar1 blocks the normal assembly of COPII at ERESs.

GTPases are involved in vesicular transport associated with a variety of signaling pathways that affect the regulation of cell proliferation and differentiation (43, 44). To further explore the role of Sar1 in the proliferation of *N. bombycis* in the present study, silkworms infected with *N. bombycis* were injected with Sar1 siRNA. After the knockdown of *NbSar1*, we found that the proliferation of *N. bombycis* was significantly inhibited (Fig. 8), which is consistent with previous research showing that the knockdown of *Sar1B* suppressed cell proliferation and induced significant apoptosis of RKO (colorectal cancer) cells (45). In Colaphellus bowringi, the knockdown of each gene of COPII considerably inhibited yolk deposition and ovarian growth. Furthermore, the silencing of *Sar1*, *Sec23*, and *Sec24* suppressed feeding and increased mortality (46). The knockdown of *Sar1b* in zebrafish embryos also caused abnormal craniofacial skeletal development and the retention of intracellular collagen (47).

It was unexpected that the expression of *NbSar1*, *NbSec23/NbSec24*, and *NbSec13/NbSec31* was significantly upregulated after interference on *NbSar1* at 96 h (Fig. 6B to F). A previous study showed that the expression of the *Rab11* gene was downregulated at 2 days whereas it exhibited upregulation at 7 days under continuous feeding of double-stranded RNA (dsRNA)-expressing bacteria or dsRNA for 14 days, which may be due to the stress response or the resistance of insects to RNAi (48). Moreover, similar results revealed that virus- or viroid-derived siRNAs are involved in the silencing of host genes and lead to the development of typical symptoms associated with the respective diseases (49–52). The host possesses RNAi mechanisms specific to the virus; accordingly, the virus could resist the RNAi of the host cell through mutation of its target gene, the generation of some viral proteins, and resistance to Dicer activity (53–55). In light of the function of Sar1 being similar to that of GTPase, we suspect that the *N. bombycis* also has a stress response or resistance to RNAi, but the mechanism remains unknown.

**FIG 9:**
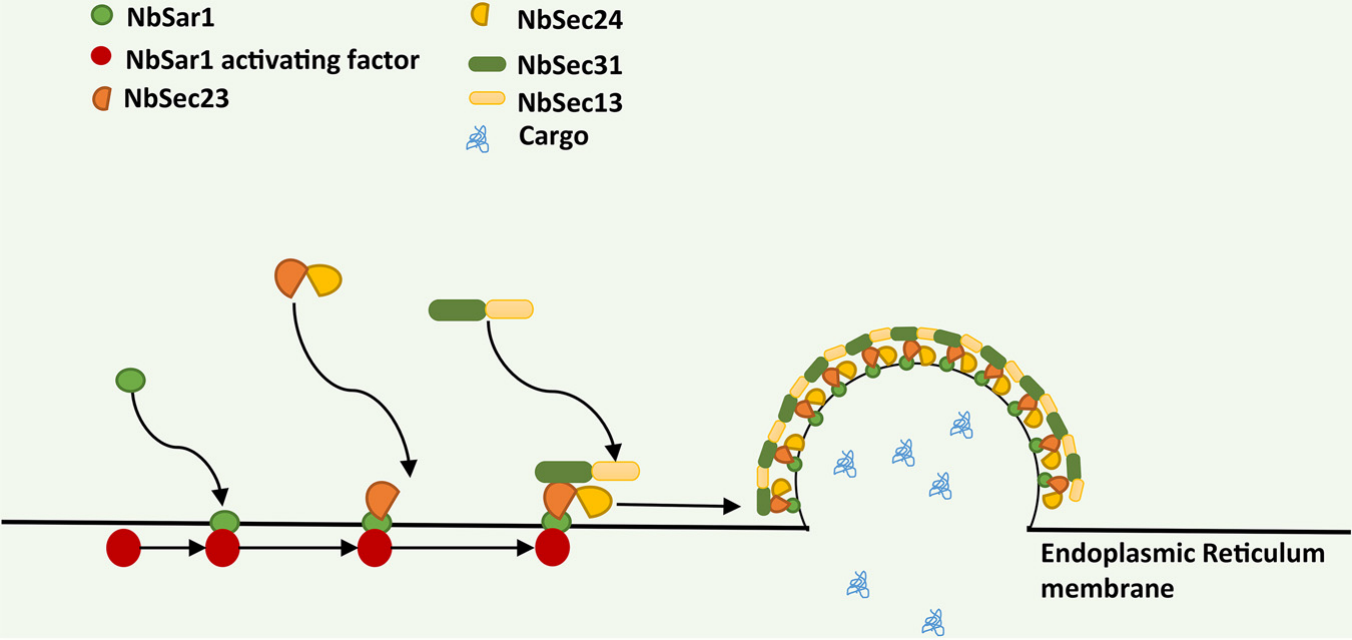
Model of COPII assembly in *N. bombycis*. NbSar1 is activated under the action of an NbSar1-activating factor (unknown at present, but we suspect that it exists), and activated NbSar1 then binds with NbSec23-2 to recruit the NbSec23-1/NbSec23-2–NbSec24 complex, thus forming the COPII inner layer. In combination with NbSec31-1, NbSec23-1 is recruited to the NbSec13–NbSec31-1 complex, thus forming the COPII outer layer.

In summary, the results of this study indicate that NbSar1 is distributed mainly in the perinuclear region; that NbSar1 interacts with NbSec23-2, Nbsec23-2 interacts with NbSec24-1/NbSec24-2, NbSec23-1 interacts with NbSec31, and NbSec31 interacts with NbSec13; and that RNAi on *NbSar1* resulted in the aberrant expression of *NbSec23*, *NbSec24*, *NbSec31*, and *NbSec13* and inhibited the proliferation of *N. bombycis*. Based on the results of this study and previous reports, we propose a model for COPII assembly in *N. bombycis* (Fig. 9). According to this model, NbSar1 is activated under the action of an NbSar1-activating factor (unknown at present, but we suspect that it exists), and the activated NbSar1 then recruits the NbSec23-NbSec24 complex through binding NbSec23, thus forming the COPII inner layer. The NbSec13–NbSec31-1 complex is recruited to the ER through interactions between NbSec23 and NbSec31, thus forming the COPII outer layer. Eventually, NbSar1, NbSec23, NbSec24, NbSec13, and NbSec31 are assembled to form COPII at the ER membrane of *N. bombycis*. The data in this study support that NbSec23, NbSec24, NbSec31, and NbSec13 can assemble to form COPII mediated by NbSar1 of *N. bombycis* and that the functions of COPII are also closely related to the proliferation of *N. bombycis*; this may be a new target for the prevention of pébrine disease of the silkworm.

## MATERIALS AND METHODS

### Parasites, hosts, and genomes.

*N. bombycis*, silkworm ovarian cell line (BmN cells), and silkworms were provided by the Department of Silkworm Physiology and Pathology of the Institute of Sericulture of the Chinese Academy of Agricultural Sciences (Zhenjiang, China). The cells were cultured in TC-100 medium (AppliChem, Germany) supplemented with 10% fetal bovine serum (FBS) (Gibco) at 26°C. *N. bombycis* genomic data (GenBank accession number ACJZ00000000.1) were obtained from MicrosporidiaDB (https://microsporidiadb.org) or GenBank.

### Cloning and expression of the *NbSar1* gene and sequence analysis.

Purified mature spores of *N. bombycis* (10^8^ spores/mL) were crushed in a tube containing 0.7 g of acid-washed glass beads for 1 min and then cooled on ice; this was repeated 10 times. The broken-spore suspension was used to extract genomic DNA according to the instructions of the fungal genomic DNA rapid extraction kit (TaKaRa, Japan). The purified genomic DNA was stored at −20°C after measuring the concentration. The forward primer 5′-GAATTCATGGAAGAAGCCAAAAAACTCTTCGG-3′, containing an EcoRI restriction site (GAATTC), and the reverse primer 5′-CGAGCTCTTATTTCTTTTGTTGGTTTATCATAATCTC-3′, containing a SacI restriction site (GAGCTC), were designed according to the sequences of the *NbSar1* gene (GenBank accession number EOB13412.1). The 50-μL PCR amplification reaction mixture contained 2 μL (200 ng) genomic DNA of *N. bombycis*, 2 μL (20 μM) each forward and reverse primers, and 25 μL PrimeSTAR HS DNA polymerase (TaKaRa, Japan), with the addition of double-distilled water (ddH_2_O) to bring the total volume to 50 μL. The PCR amplification products were separated by 1% agarose gel electrophoresis and recovered using the AxyPrep DNA gel extraction kit (TaKaRa, Japan). The recovered products were cloned into the pMD19-T vector (Axygen, USA). The recombinant vector pMD19-T-*NbSar1* was transformed into Escherichia coli Top10 competent cells (Sangon Biotech, China) and then cultured on LB plates containing ampicillin. The recombinants were identified by PCR and sequenced by Sangon Biotech (Shanghai, China). pMD19-T-*NbSar1* was digested by EcoRI and SacI, and the *NbSar1* gene was then ligated into the expression vector pET-28a. The pET-28a-*NbSar1* recombinant plasmid was transformed into E. coli BL21(DE3) strain cells (Sangon Biotech, Shanghai, China) and cultured on LB plates containing kanamycin. Single colonies were selected for PCR identification and sequencing (Sangon Biotech, Shanghai, China). Recombinant pET-28a-*NbSar1* was induced with 0.5 mM IPTG (isopropyl-β-d-thiogalactopyranoside) for 4 h. After cultivation, bacterial cells were collected and destroyed by sonication. SDS-PAGE was performed to confirm the expression of the recombinant protein, and the recombinant protein was tested by Western blotting using His tag antibody (Sangon Biotech, Shanghai, China). The expressed recombinant proteins were purified with the HisTrap FF purification kit (GE Healthcare, USA) according to the manufacturer’s instructions.

The isoelectric point (pI) and molecular weight of NbSar1 were predicted by the ExPASy Compute pI/Mw tool (https://web.expasy.org/compute_pi/). The homologous sequences were compared by using DNAMAN.

### Preparation of NbSar1 polyclonal antibody and Western blot analysis.

New Zealand White rabbits were injected with 300 μg of purified recombinant protein emulsified with Freund’s complete adjuvant one time (Sigma, Germany) and then injected subcutaneously with 100 μg of purified recombinant protein emulsified with Freund’s incomplete adjuvant four times within 2 months. A small amount of rabbit antiserum was collected after immunization every time, and the antibody titer of the rabbit serum was assessed more than 1:8 0000 using enzyme-linked immunosorbent assay (ELISA). The antiserum was collected after the final immunization and purified by antigen affinity purification. Preimmune serum of rabbits was screened to confirm the absence of endogenous antibody that reacted with NbSar1. The animal experiment was carried out in Shanghai Youke Biotechnology Co., LTD. The animal Use license (SYXK(Shanghai)2018-0020) was approved by Shanghai Commission of Science and Technology (SCST).

The spore suspension broken by acid-washed glass beads was used to extract the total protein of *N. bombycis*. NbSar1 in the total protein was tested by Western blotting using the NbSar1 antibody.

### Immunolocalization of NbSar1 in *N. bombycis*.

The germination of mature spores of *N. bombycis* was performed in 0.2 M KOH at 27°C for 1 h. BmN cells were cultured to a density of 80% in six-well plates. The cells were inoculated with the germinating spores in six-well plates and then cultured for 7 days. The cells infected with *N. bombycis* were fixed with 4% paraformaldehyde for 1 h, washed 3 times in phosphate-buffered saline–Tween (PBST) for 5 min, and subjected to permeabilization with 0.1% Triton X-100 for 1 h at room temperature. After being washed 3 times in PBST for 5 min, the cell samples were blocked with 5% bovine serum albumin (BSA) at 4°C overnight. The cell samples were incubated with 1 μg/mL NbSar1 antibody for 1 h at room temperature, washed 3 times in PBST for 10 min, and incubated in 1 μg/mL Alexa Fluor 488-conjugated goat anti-rabbit IgG (Sangon Biotech, Shanghai, China) at room temperature for 1 h successively. After being washed 3 times in PBST for 10 min, the cell samples were incubated with 2 μg/mL *N. bombycis* actin antibody (provided by the Department of Silkworm Physiology and Pathology of the Institute of Sericulture of the Chinese Academy of Agricultural Sciences, China) for 1 h, washed 3 times in PBST for 10 min, incubated with 5 μg/mL Cy5-conjugated goat anti-rabbit IgG (Sangon Biotech, Shanghai, China) for 1 h, and washed 3 times in PBST for 5 min. The nuclei were stained with 4′,6-diamidino-2-phenylindole (DAPI) for 20 min at room temperature. Negative controls were treated with preimmune serum as the primary antibody. Immunolocalization was observed by using an inverted fluorescence microscope (Olympus IX-71).

### Yeast two-hybrid assay.

*NbSar1*, *NbSec23-1*, *NbSec23-2*, *NbSec24-1*, *NbSec24-2*, *NbSec13*, *NbSec31-1*, and *NbSec31-2* were amplified from genomic DNA of *N. bombycis* using specific primers (Table 1), and *NbSar1* digested by EcoRI and NotI was ligated into the bait vector pGBKT7-BK, while *NbSec31-1* and *NbSec24-2* were ligated into the yeast two-hybrid bait vector pGBKT7-BK using an In-Fusion HD cloning kit (TaKaRa, Japan). *NbSec13* and *NbSec23-1* digested by EcoRI and XhoI were ligated into prey vector pGADT7-AD, while *NbSec24-1* digested by EcoRI and NotI was ligated into prey vector pGBKT7-BK, and *NbSec23-2* was ligated into prey vector pGADT7-AD using an In-Fusion HD cloning kit (TaKaRa, Japan). The bait recombinant vector and prey recombinant vector were cotransformed into Y2H Gold yeast cells (Weidi Biotech, Shanghai, China) and then cultured on SD−Leu/−Trp plates (Clontech, USA) at 28°C. A single colony on SD−Leu/−Trp plates was selected and cultured on SD−Ade/−His/−Leu/−Trp plates containing X-α-gal (Clontech, USA) at 28°C.

**TABLE 1:** Primer sequences for PCR

Gene	Primer direction	Primer sequence (5′–3′)
*NbSar1*	Forward	CCGGAATTCATGGAAGAAGCCAAAAAA
	Reverse	TTGCGGCCGCTTATTTCTTTTGTTGGTTTAT

*NbSec23-1*	Forward	CCGGAATTCATGGCTTACTATCCAAATTTTATG
	Reverse	CCGCTCGAGTTATTCATCACTACTCACAACAA

*NbSec23-2*	Forward	GGAGGCCAGTGAATTCATGGAAGAAGCAATCAGAGAAATCG
	Reverse	TCATCTGCAGCTCGAGCTGCAGGTCATATTCCTCATAAG

*NbSec24-1*	Forward	CCGGAATTCATGACAGACGATTCTTCTCAAGTTTA
	Reverse	TTGCGGCCGCCTACCACCACACTTCCCTTACATTA

*NbSec24-2*	Forward	CATGGAGGCCGAATTCATGTTTAAGATTGTCCTTAAAAGT
	Reverse	GCTAGTTATGCGGCCGCTTATGTTGATGATCCTCGTACAAA

*NbSec13*	Forward	CCGGAATTCATGGAGTCCCATAAAATCCAC
	Reverse	CCGCTCGAGTTATTCACCACATTTACTAAG

*NbSec31-1*	Forward	CGCCATATGATGATAAGTCTTTATGAACCCG
	Reverse	TTGCGGCCGCTTAATAAACTAATTGAACCAGTGT

*NbSec31-2*	Forward	CGCCATATGATGAAAATCAACAAAAGGTG
	Reverse	TTGCGGCCGCTTAATAAACTAATTGGACCAGTG

### RNAi of NbSar1.

Small interfering RNA (siRNA) was designed according to the sequence of the *NbSar1* gene and synthesized by Sangon Biotech (Shanghai, China). Fresh mulberry leaves were smeared with a 10^8^-spore/mL suspension of *N. bombycis*, and P50 silkworms of the fifth instar were fed on the mulberry leaves smeared with *N. bombycis*. After feeding for 6 h, each silkworm was injected with 3 μL of siRNA (Table 2). After the injection of siRNA at 24 h, 48 h, 72 h, and 96 h, the midguts of the silkworms were collected and kept at −80°C. The midgut was lysed with 1 mL of RNAiso plus lysate (TaKaRa, Japan), the total RNA was then extracted with a Mini Best universal RNA extraction kit (TaKaRa, Japan), and cDNA was synthesized with PrimeScript RT master mix (TaKaRa, Japan). cDNA was used as the template to perform qPCR using the TB green premix Ex *Taq* II (*Tli* RNase H Plus) kit (TaKaRa, Japan) according to the manufacturer’s instructions, and the primer sequences are shown in Table 2. The transcription levels were calculated by the 2^−ΔΔ^*^CT^* method with three replicates. GraphPad Prism 7.0 (GraphPad Software, San Diego, CA, USA) was used to conduct the multiple *t* tests.

**TABLE 2:** Primer sequences for RNAi and qPCR

Gene (purpose of primer)	Primer direction	Primer sequence (5′–3′)
*NbSar1* (RNAi)	Sense	CCAAGUCCCAAAUUGAAAUTT
	Antisense	AUUUCAAUUUGGGACUUGGTT

Negative control (RNAi)	Sense	UUCUCCGAACGUGUCACGUTT
	Antisense	ACGUGACACGUUCGGAGAATT

*NbSar1* (qPCR)	Forward	AATGCGGGTAAGACTACTCTGG
	Reverse	CAAGCAACTCGAGCTGCTTTGTG

*N. bombycis* β-tubulin (qPCR)	Forward	TTCCCTTCCCTAGACTTCACTTC
	Reverse	CAGCAGCCACAGTCAAATACC

*NbSec23-1* (qPCR)	Forward	GCGAAAGAAATCCAACTCCTC
	Reverse	CGTAATCACTGTCCCACTAGAA

*NbSec24-1* (qPCR)	Forward	GACAGACGATTCTTCTCAAGTTTATTC
	Reverse	TCTTCATTATAATTCTCGGGTTGGA

*NbSec24-2* (qPCR)	Forward	CCTTTCCTAATTTCTATCCTCCTCAT
	Reverse	CCTTATCATTCTCTGCCCTTTCT

*NbSec13* (qPCR)	Forward	GTGGAGGTGTGTCCTGATAATG
	Reverse	AGAGAGACTATAGACGGGTTCAC

*NbSec31-1* (qPCR)	Forward	TCCACGAGTAGTTCCGAGTATG
	Reverse	CGGGTACAGAATCCACTTGATG

*NbSec31-2* (qPCR)	Forward	CACGTGTAGTTCCGAGTATGAG
	Reverse	TACAGGAGGCCTAGTACAGAAT

*BmGAPDH* (qPCR)	Forward	TTCATGCCACAACTGCTACA
	Reverse	AGTCAGCTTGCCATTAAGAG
