# Supplementary material for: Sar1 Interacts with Sec23/Sec24 and Sec13/Sec31 Complexes: Insight into Its Involvement in the Assembly of Coat Protein Complex II in the Microsporidian Nosema bombycis
Source: Microbiol Spectr. 2022 Oct 27;10(6):e00719-22. doi: 10.1128/spectrum.00719-22 (PMC9769691; doi:10.1128/spectrum.00719-22)
Supplement: Supplemental file 1 — Supplemental material. Download spectrum.00719-22-s0001.pdf, PDF file, 1.1 MB [file spectrum.00719-22-s0001.pdf]

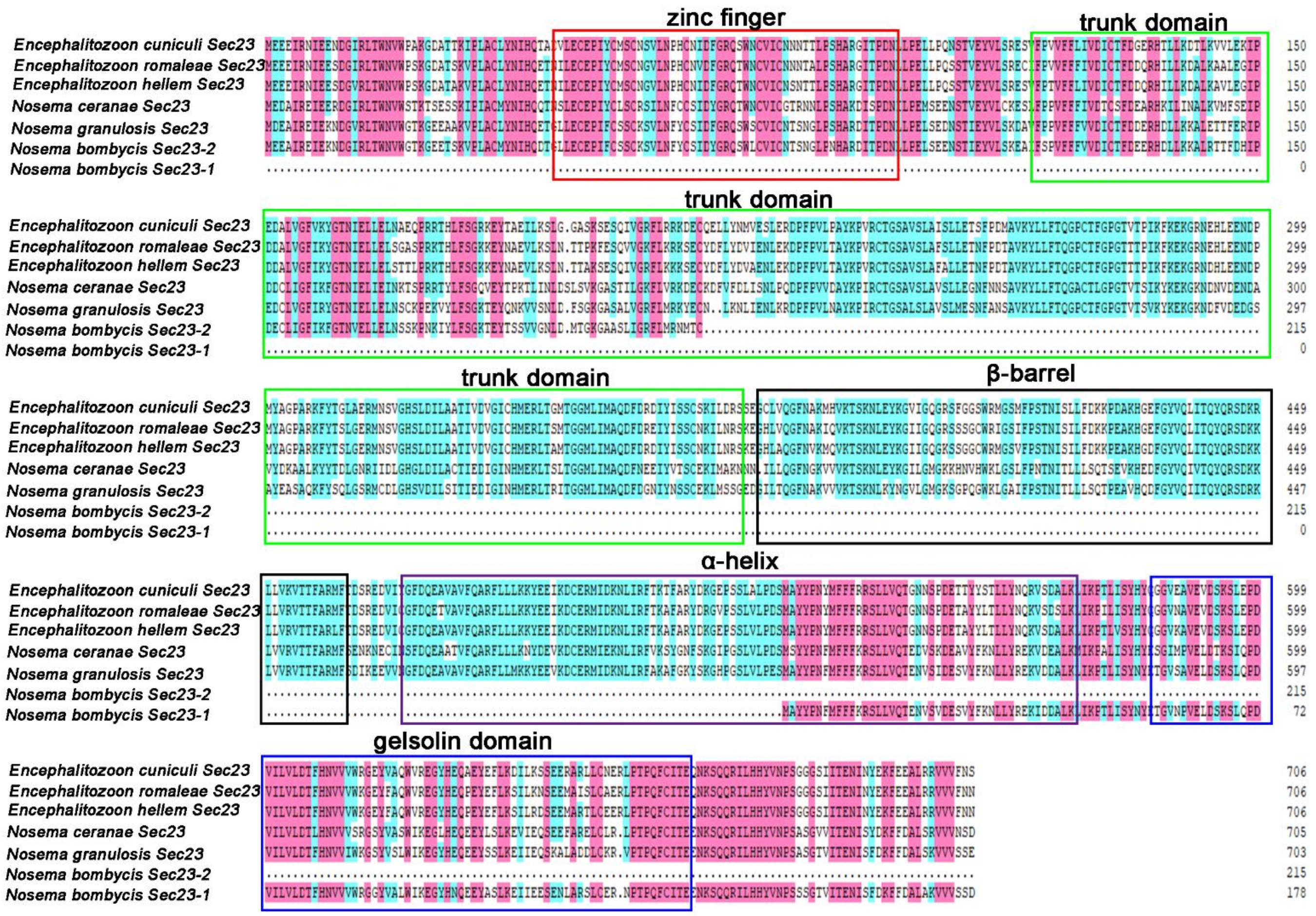

Fig. S1 Amino acid alignment of Sec23 from microsporidian species. The red box showed the zinx finger, the green box showed the trunk domain, the black box showed the β-barrel, the purple box showed the α-helix, the blue box showed the gelsolin domain.

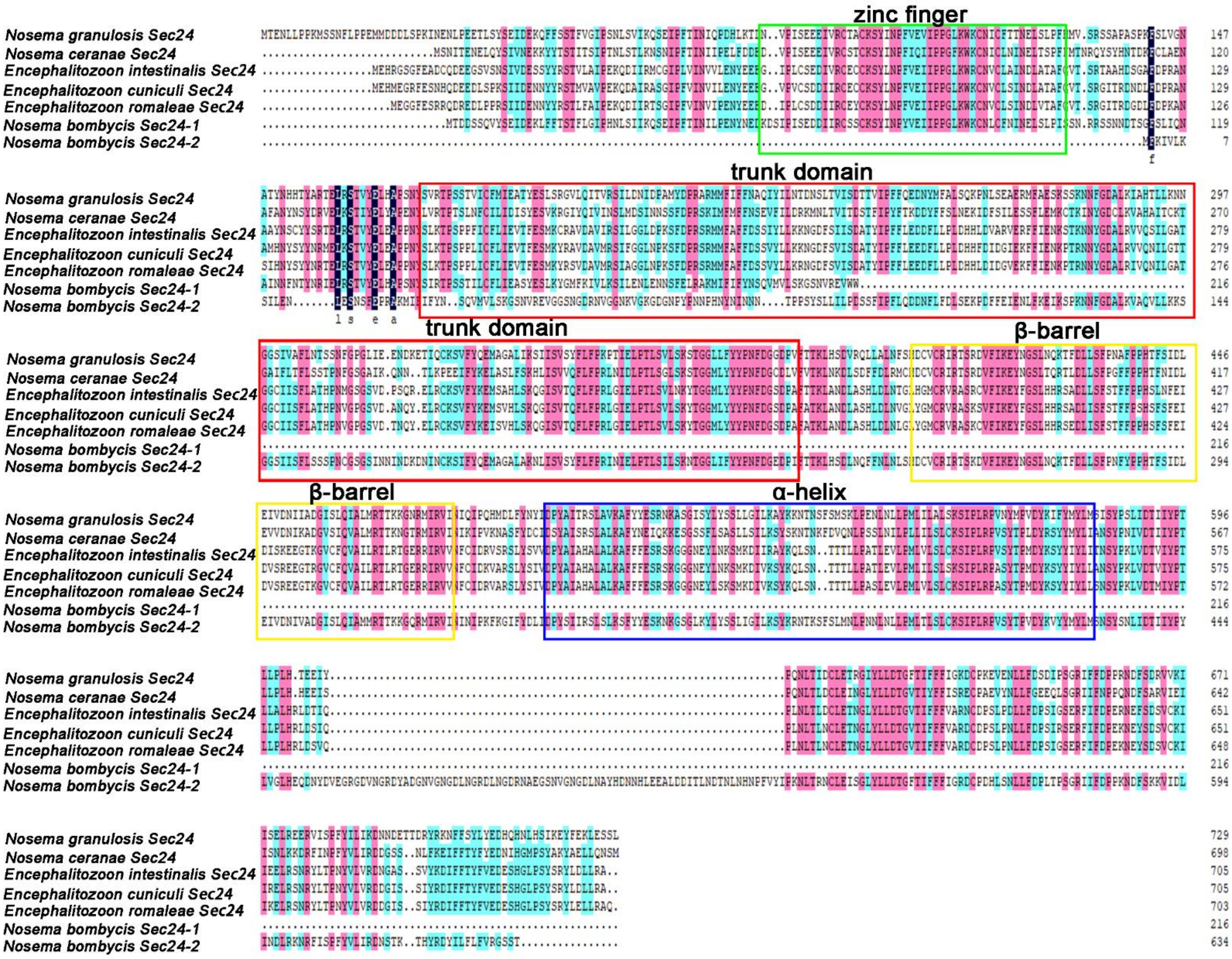

Fig. S2 Amino acid alignment of Sec24 from microsporidian species. The green box showed the zinx finger, the red box showed the trunk domain, the yellow box showed the β-barrel, the blue box showed the α-helix.
